# Developmental-Centered Care in Preterm Newborns: Scoping Review

**DOI:** 10.3390/children12060783

**Published:** 2025-06-16

**Authors:** Jina M. Velasco Arias, Aida M. Peres, Francisco M. Escandell Rico, M. Carmen Solano-Ruiz, Vicente F. Gil-Guillen, Ana Noreña-Peña

**Affiliations:** 1 Postgraduate Program in Nursing, Health Sciences Sector, Federal University of Paraná, Curitiba 80060, PR, Brazil; jina.velasco@correounivalle.edu.co (J.M.V.A.); amaris@ufpr.br (A.M.P.); 2 Department of Nursing, Faculty of Health Sciences, University of Alicante, 03080 Alicante, Spain; carmen.solano@ua.es (M.C.S.-R.); ana.norena@ua.es (A.N.-P.); 3 Network for Research on Chronicity, Primary Care and Health Promotion (RICAPPS), 03080 Alicante, Spain; vgil@umh.es; 4 Clinical Medicine Department, School of Medicine, Miguel Hernandez University, 03202 Alicante, Spain

**Keywords:** infant, premature, child development, intensive care units, neonatal, kangaroo mother care method

## Abstract

**Background/Objectives:** Preterm newborns often require specialized care and management. However, exposure to multiple stimuli during hospitalization can adversely affect their neurological development. Developmental-centered care integrates evidence-based practices and neuroprotective strategies to create an optimal care environment that minimizes harmful stimuli in the neonatal intensive care unit (NICU) and supports the neurological development of preterm infants. To identify interventions related to developmental-centered care that support preterm newborns in the NICU. **Methods:** A scoping review was conducted following the guidelines of the Joanna Briggs Institute and the Preferred Reporting Items for Systematic Reviews and Meta-Analyses extension for scoping reviews. A comprehensive search was performed in MEDLINE, CINAHL, and Web of Science databases. The results were categorized based on the general characteristics of the studies and the main interventions related to developmental-centered care. **Results:** Out of 163 potentially relevant sources identified, 19 studies met the inclusion criteria. A total of 52 interventions were identified and classified into three thematic categories related to the benefits of the kangaroo care method, the family-centered care model, and the neonatal individualized developmental care and assessment program. **Conclusions:** The findings underscore the importance of integrating developmental-centered care practices, such as skin-to-skin contact, specialized feeding, and active parental involvement, into clinical practice to support neurodevelopment and improve health outcomes in preterm infants.

## 1. Introduction

According to the World Health Organization (WHO), a preterm newborn (PN) is defined as an infant born before completing 37 weeks of gestation. It is estimated that approximately 15 million PNs are born each year, accounting for about 10% of all births worldwide [1]. Between 24 and 40 weeks of gestation, the fetal nervous system undergoes a critical period of development and maturation that is essential for proper neuromotor and behavioral functioning in the newborn [2].

In PNs, the immaturity of organ systems resulting from the interruption of gestation predisposes them to complications, such as growth delay, hearing loss, visual impairment, and the premature onset of chronic diseases [3,4]. Consequently, PNs often require admission to the neonatal intensive care unit (NICU) for specialized care and management. However, exposure to multiple stimuli in the NICU, such as excessive noise, artificial light, and painful medical procedures, may affect the neurological development of PNs differently than if they were in the protective environment of the uterus. This may lead to developmental differences compared to full-term newborns [2,5].

Excessive sensory stimulation in the NICU can adversely affect the development of motor skills and neurobehavioral traits in PNs later in life [6]. Approximately 25% of these infants experience developmental disorders by the age of 2, with this figure rising to 40% by age 10 [2]. In addition, family separation during care negatively impacts the emotional development of PNs by disrupting the mother–infant bond [7].

In recent decades, the increased survival rate of PNs in the NICU has spurred research interest in how the hospital environment affects both their neurological development and their relationship with their families. This growing interest has gradually fostered the advancement of developmental care practices that promote active parental involvement and strengthen the parent–infant bond, such as the family-centered care model (FCCM) [7,8].

In this context, the integrative developmental care model (IDCM), grounded in developmental-centered care (DCC), has also emerged. DCC incorporates evidence-based best practices aimed at enhancing neuroprotective strategies for PNs [9,10]. As a result, a wide array of strategies has been developed to minimize the environmental stress experienced by PNs in the NICU [7].

DCC encompasses strategies such as noise reduction management [11], the kangaroo care method (KCM), which promotes skin-to-skin contact between the PNs and their parents [12,13], and the neonatal individualized developmental care and assessment program (NIDCAP) [14]. These strategies, inspired by the intrauterine environment, contribute to establishing a therapeutic setting designed to minimize pain, protect the skin, optimize nutrition, and enhance family involvement [15].

Both the FCCM and the DCC approach have demonstrated multiple benefits. These strategies not only enhance the experience of preterm newborns (PNs) during their NICU stay but also promote neurodevelopment, facilitate a faster and safer recovery, provide greater emotional support to parents, and reduce hospital costs [6,16].

Prematurity is a leading cause of neonatal mortality, accounting for more than half of the 2.3 million neonatal deaths globally each year. To address this issue, the Sustainable Development Goals (SDGs) include a target under Goal 3.2 to reduce the neonatal mortality rate to 12 or fewer deaths per 1000 live births by 2030 [17]. In this context, it is essential for healthcare professionals to identify strategies that effectively mitigate the global burden of complications in preterm neonates, thereby advancing progress toward achieving the SDGs [18].

Furthermore, there is growing evidence that providing high-quality interventions during the early years of life not only helps reduce health inequities but also enhances children’s learning and academic outcomes, while substantially improving health and economic productivity in adulthood [19]. This evidence underscores the importance of implementing effective interventions during critical developmental periods across the lifespan, with a particular emphasis on early childhood [18].

Therefore, the aim of this study is to identify developmental-centered care interventions for managing preterm neonates in the NICU. In this regard, an analysis of the scientific evidence can provide insights into which interventions promote neuroprotection and yield developmental benefits for PNs when applied during hospitalization.

## 2. Materials and Methods

### 2.1. Study Design

This scoping review of the literature follows the guidelines established by the Joanna Briggs Institute (JBI) [20] and the Preferred Reporting Items for Systematic Reviews Extension for Scoping Reviews (PRISMA-ScR) [21]. A scoping review facilitates the identification of key concepts underpinning a field of study and clarifies the operational definitions or conceptual boundaries of a specific topic. This methodology enables a comprehensive and systematic literature synthesis and helps establish a foundational knowledge base for future empirical studies [21]. No protocol was registered for this review.

### 2.2. Identification of the Topic and Research Question

The research question was developed using the PCC acronym, which includes the following elements: population (P), referring to premature newborns; concept (C), referring to developmental care; and context (C), within the neonatal intensive care unit [20]. Based on this framework, the following question was formulated: Which developmental care interventions are implemented in the care of premature newborns in the neonatal intensive care unit?

### 2.3. Eligibility Criteria

According to the PCC framework, articles were included in this review if they met the following criteria: (1) peer-reviewed primary studies; (2) published between 2014 and 2024; (3) written in English, Portuguese, or Spanish. A ten-year search period was selected in accordance with recommended practices for literature reviews, with the aim of encompassing relevant publications on the subject of study. Regarding study design, articles employing quantitative, qualitative, and mixed-methods approaches were considered eligible.

Articles were excluded if they: (1) did not address the DCC; (2) focused on neuroprotective strategies applied outside the NICU; (3) were centered on specific pathologies, palliative care, or surgical interventions; or (4) failed to meet quality criteria, such as lacking clear specification of the studied population, study design, or other relevant methodological aspects.

### 2.4. Information Sources

Studies were identified through a comprehensive search conducted in September 2024 across three databases: Medical Literature Analysis and Retrieval System Online (MEDLINE), Cumulative Index to Nursing and Allied Health Literature (CINAHL), and Web of Science (WOS). Furthermore, to conduct a more comprehensive search and obtain broader results, an update search was performed on these same databases in December 2024.

The search strategy employed the following medical subject heading (MeSH) keywords: “newborn”, “preterm”, “premature”, and “kangaroo mother care method”. Additionally, other natural language terms were included, such as “NIDCAP”, “newborn individualized developmental care and assessment program”, “neuroprotective measure”, “developmental care”, “neonatal developmental care”, “family-centered care”, “neonatal intensive care unit”, and “NICU”. All terms were adapted to the specific requirements of each database, using Boolean operators ANDand OR.

### 2.5. Selection of Sources of Evidence

The study selection process was conducted using the online platform Rayyan, which is designed to streamline systematic reviews [22]. Initially, duplicate articles were removed, and the titles and all abstracts of all references identified through the search strategy were screened. Two authors (JMV and ANP) independently evaluated the references, and selected studies deemed potentially eligible based on the PCC criteria.

Next, the remaining authors conducted a detailed review of the full texts to confirm eligibility. Studies meeting the inclusion criteria were retrieved in full and independently re-evaluated by each participating researcher. Any discrepancies were resolved through consensus among all team members, resulting in a consensus rate of 90%. Studies lacking agreement were excluded.

After the final selection of articles for the review, three researchers (JMV, AM, and FME) independently carried out the subsequent processes in several phases: screening, consensus-building, and result verification. The Mendeley Reference Manager was used to facilitate the organization and management of the selected documents.

### 2.6. Data Extraction and Result Synthesis

Data extraction was performed using a customized form developed in Microsoft Excel^®^ (2021), in accordance with the methodological guidelines for scoping reviews outlined by JBI [23]. The form was designed to map the scope and systematically document the characteristics of each study. Extracted data included the title, authors, publication year, language, study location, objective, study design, level of evidence, sample size, descriptions of interventions related to DCC, and study conclusions.

While assessing the quality and level of evidence is not a standard practice in scoping reviews [23], this step was included given that only peer-reviewed studies were considered. For this purpose, the framework proposed by Melnyk and Fineout-Overholt [24] was employed. This assessment aimed to provide a more comprehensive understanding of the quality of the evidence included in the review. The data on DCC interventions were categorized into four sections: (1) general characteristics of the studies; (2) interventions involving DCC for PNs in the NICU; (3) benefits of the FCCM for parents and PNs in the NICU; (4) benefits of the NIDCAP for parents and PNs in the NICU.

## 3. Results

**Figure 1:**
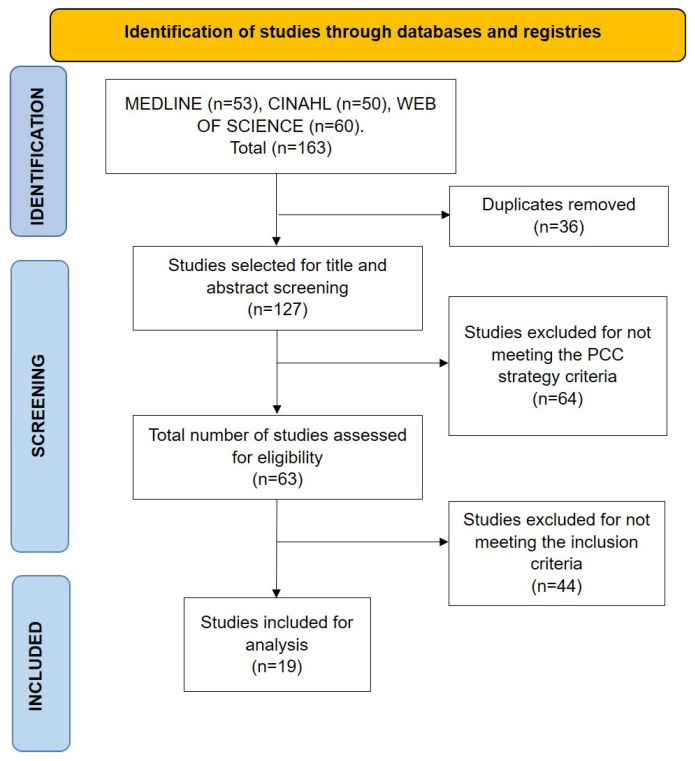
Flowchart based on the PRISMA guidelines [23].

The search identified 163 potentially eligible studies. After removing duplicate references, 127 studies remained. Of these, 64 articles were excluded for not meeting the PCC criteria, confirming the expectation that the search strategy would retrieve a substantial proportion of irrelevant records. Consequently, 63 studies were selected for full-text review, and 19 articles ultimately met the inclusion criteria to address the objective of this review, as detailed in Figure 1.

### 3.1. General Characteristics of the Studies

This review analyzed 19 primary studies published in English between 2014 and 2024, encompassing a total of 2819 participants from 12 countries. The United States accounted for the highest number of studies (n = 3), followed by India, China, and Iran (n = 2 each). All studies focused on PNs hospitalized in NICUs, with gestational ages ranging from 22 to 36 weeks. Regarding study design, randomized controlled trials (RCTs) were the most prevalent, comprising 36.8% (n = 7) of the included studies. The sample sizes ranged from 20 to 718 PNs.

All interventions were related to DCC and were implemented in NICUs throughout the hospitalization period until discharge. Table 1 provides a summary of the general characteristics of each study.

A review of the articles identified the primary strategies related to DCC for PNs, including KCM [25,28,31,32,34,35,36,37,38,39,40,41], the FCCM [26,29,30,33,42,43], and the NIDCAP [27]. These strategies emphasize interventions such as skin-to-skin contact, breastfeeding, pain and stress management through the clustering of procedures, appropriate infant positioning, reduction of environmental stimuli in the NICU, active parental involvement in newborn care, and healthcare team education. Table 2 provides a summary of the specific interventions described in the articles.

### 3.2. Benefits of the Kangaroo Care Method for the Development of PNs in the NICU

The KCM is based on skin-to-skin contact between the infant and their parents through proper positioning, which enhances physiological stability and neurobehavioral development. This close interaction fosters a stronger emotional bond between parents and the PN, while also supporting exclusive breastfeeding, which is crucial for ensuring adequate nutrition [35,38,39,40].

Although KCM is typically provided by the mother, the father can also assume this role, and no significant differences in physiological responses or stress levels have been observed between maternal kangaroo care (MKC) and paternal kangaroo care (PKC). Both approaches are equally effective and safe, offering similar benefits for the newborn’s stability [34,41].

Among the benefits identified, Chaudhari et al. [39] highlight that KCM improves cerebral blood flow and stabilizes cardiorespiratory parameters in PNs. Similarly, a controlled clinical trial conducted by El-Farrash et al. [40] found that a longer duration of KCM enhances neurobehavioral performance and optimizes both thermal regulation and tissue oxygenation.

Furthermore, El-Farrash et al. [40] emphasize that KCM not only promotes breastfeeding but also accelerates the transition to full enteral feeding. While moderate to high levels of breast milk intake may not significantly impact the behavioral skills of PNs, Gao et al. [28] suggest that it contributes to the motor and neurological development in extremely preterm infants and reduces the risk of intraventricular hemorrhage.

A prospective cohort study conducted by Pineda et al. [31] suggests that increased parental–infant contact in the NICU is associated with enhanced neurobehavioral development in PNs prior to hospital discharge. Furthermore, an increase in skin-to-skin contact during the NICU stay was linked to better development of both gross and fine motor skills at 4 and 5 years of age [31].

Skin-to-skin contact is typically performed with the infant positioned on the caregiver’s chest; however, this technique can also be implemented with the infant on the caregiver’s back, without compromising its benefits [36]. Nevertheless, Buil et al. [38] highlight that supported diagonal flexion (SDF) positioning, in which the infant is placed in a supine position with the feet turned to one side, provides greater opportunities for communication between the mother and the infant, fostering a higher-quality interaction between them.

Conversely, the benefits of skin-to-skin contact are further amplified when combined with other interventions, such as music therapy. A clinical trial conducted by Yakobson et al. [35] revealed that this combination enhances autonomic nervous system stability in PNs. This improvement in autonomic regulation significantly contributes to the recovery and maturation processes of neonates [35].

However, Brignoni-Pérez et al. [25] note that certain factors can affect the benefits of KCM. Aspects such as low socioeconomic status or language barriers between parents and healthcare providers may limit parental involvement in this practice, impacting the rate, frequency, and duration of KCM experienced by PNs in the NICU [25].

Despite the availability of artificial devices, such as mattresses designed to simulate skin-to-skin contact, the benefits observed are not comparable to those achieved when this contact is provided directly by the parents [32]. Nevertheless, in the absence of the parents, KCM provided by a surrogate mother has proven to be equally effective in improving arterial oxygen saturation and feeding in preterm neonates [37].

On the other hand, Pavlyshyn et al. [29] emphasize that modifying the environment and combining interventions to reduce stress and pain contribute to improved clinical outcomes in PNs. When coupled with active parental involvement, these practices help lower the incidence of late-onset sepsis, retinopathy of prematurity, periventricular leukomalacia, and feeding intolerance. Furthermore, they contribute to a shorter duration of ventilatory support, reduced antibiotic use, and decreased reliance on parenteral nutrition, resulting in improved weight gain [29].

### 3.3. Benefits of the FCCM for Parents and PNs in the NICU

DCCs are closely associated with the FCCM, which fosters collaboration between the healthcare team and the parents of PNs by actively engaging them in their infants’ care [30,33,42]. While care for PNs is primarily provided by healthcare professionals, the FCCM enables parents to participate in daily medical rounds. In addition, parents receive education on infant care and development, hand hygiene, breastfeeding, and the importance of skin-to-skin contact, all of which contribute to improved clinical outcomes [30].

Compared to standard care, FCCM actively involves parents and families in planning of care for PNs. This approach includes interventions such as education and psychosocial support for parents, which facilitate effective communication with the healthcare team. As a result, PNs experience better neurobehavioral outcomes, and the length of stay in the NICU is reduced [33,42].

Some countries have begun adapting the FCCM to address their specific needs. For example, in Canada, the Alberta Family Integrated Care (FICare) model supports parents in caring for their babies in level II NICUs, facilitating earlier discharge [43]. According to Benzies et al. [43], implementing this model has successfully reduced the length of hospital stay for preterm infants between 32 and 34 weeks of gestation, without increasing readmissions or emergency room visits.

In the United States, the implementation of an enhanced FICare program incorporating mobile technology in the NICU revealed that PNs whose parents actively participated in the program experienced a significant increase in weight gain and fewer hospital-acquired infections. These findings underscore the need to improve hospital data systems to better capture information regarding parental presence and involvement in the care of PNs [26].

### 3.4. Benefits of the NIDCAP for Parents and PNs in the NICU

The NIDCAP has proven to be an important tool for the neurodevelopmental care of PNs. Saldanha and Tauro [27] assessed the effectiveness of NIDCAP interventions during the hospitalization of PNs in the NICU. These interventions included training for parents in areas such as communication and security, feeding, positioning, kangaroo care infection prevention, and skin care.

In their study, Saldanha and Tauro [27] observed an improvement in mothers’ competencies in caring for their newborns, along with enhanced clinical outcomes for PNs, such as better oxygenation and increased weight gain. These findings suggest that educating parents on interventions based on the NIDCAP approach not only boosts their confidence but also reduces the risk of neonatal readmissions following hospital discharge [27].

## 4. Discussion

This review identified 19 studies that examined various interventions related to DCC in different countries, as well as their effects on PNs and their families. The interventions were categorized into three main strategies: the kangaroo care method [25,28,31,32,34,35,36,37,38,39,40,41], family-centered care model [26,29,30,33,42,43], and the NIDCAP approach [27]. Overall, the findings from this review highlight the positive impact of implementing DCC in the care of hospitalized PNs in the NICU.

DCC comprises a set of interventions focused on interpreting newborn behavior and implementing strategies to minimize stress, particularly during hospitalization in the NICU. These interventions aim to promote both the neurological and emotional development of the newborn while enhancing the active involvement of the family in the infant’s care [44].

A systematic meta-analysis by Soleimani et al. [2] found that DCC interventions in the NICU significantly improved both the mental development index and the psychomotor development index at 12 months corrected age, with standardized mean differences of 0.55 and 0.33, respectively. These benefits are even more pronounced when PNs are cared for in single rooms within the NICU, compared to those treated in an open-bay NICU [45].

Among the interventions related to DCC, the WHO highlights KCM as one of the most effective strategies for reducing mortality in preterm and low birth weight infants [46]. This approach not only reduces the incidence of severe infections or sepsis but also increases the duration of exclusive breastfeeding at discharge, thereby contributing to improved growth and development of the infant [47].

Beyond enhancing the survival rates of preterm infants, the KCM also yields long-term positive effects on social and behavioral outcomes. Children who received this type of care tend to exhibit lower rates of school absenteeism, hyperactivity, aggression, and behavioral issues. Moreover, in adulthood, these individuals demonstrate improved cognitive function in areas related to intelligence, attention, memory, and coordination [47].

The findings of this review support this perspective, highlighting the benefits for the nervous system of PNs, such as improved cerebral blood flow and tissue oxygenation, when KCM is implemented in the NICU [39,40]. Furthermore, the positive impact of KCM on neurodevelopmental outcomes in these infants is consistently reported across diverse regions of the world [28,31,34,35,37,41].

Another neurodevelopmental strategy identified in this review is FCCM. This model is founded on active collaboration between families and the healthcare team in the NICU, fostering parental involvement in the direct care of newborns. The FCCM approach has been shown to enhance the well-being of PNs and their parents by providing more humanized care that is tailored to the specific needs of each family [48].

An integrative review conducted by Gómez-Cantarino et al. [4] examined the benefits of the FCCM for PNs hospitalized in the NICU. The review found that this approach promotes parental empowerment by enabling parents to assume the role of primary caregivers, which reduces anxiety and stress while increasing their sense of security and control over the caregiving process. Active family involvement in newborn care enhances the quality of care and may reduce the duration of hospital stays. Furthermore, the FCCM facilitates better adaptation of PNs to the extrauterine environment, supporting the regulation of heart rate, respiratory rate, and temperature, while also promoting increased weight gain [4].

On the other hand, the NIDCAP stands out as an effective strategy for implementing neuroprotective interventions, as it is designed to seamlessly integrate all neuroprotective measures within the framework of the FCCM [49]. This program relies on careful observation of preterm infants’ behavior to guide care interventions, recognizing both the infant and their family as a cohesive unit [4].

The NIDCAP is implemented through a collaborative approach within the NICU, using a personalized care protocol tailored to each infant’s specific needs. These interventions, collectively known as individualized developmentally supportive care [44], include key components such as pain management, skin-to-skin contact, and active family involvement in the child’s care [49].

The individualized care approach promoted by NIDCAP enhances parental integration into the care process and allows for the adjustment of interventions based on the developmental stage of the PN, ultimately improving the quality of care within the NICU [4,50].

Despite the documented benefits of DCC for PNs, several barriers may limit its implementation. For instance, socioeconomic status has been linked to lower parental involvement in NICU activities, suggesting that a family’s economic conditions significantly affect their capacity to engage in their child’s care [51]. Additionally, language barriers can hinder the implementation of DCC. The lack of effective translation services complicates communication between non-native parents and healthcare providers, potentially leading to misunderstandings and reduced parental participation in the care of the PN in the NICU [52].

Other factors, such as insufficient institutional support and a lack of adequately trained healthcare staff, can also limit the integration of DCC into daily NICU practice [53]. For example, the implementation of NIDCAP requires significant organizational investments, including comprehensive staff education and a redefinition of roles and interactions among healthcare professionals, the infant, and the family. Without a well-established institutional protocol and a holistic care philosophy, these challenges may hinder the successful implementation of NIDCAP [44,54].

In certain cultural contexts, prevailing norms and beliefs may influence the perception and acceptance of DCC interventions, thereby impacting their implementation [55]. In China, barriers such as cultural resistance from grandparents and the costs associated with caregiver accommodation have been identified, hindering the widespread adoption of DCC [56].

Conversely, traditional NICU care models often fail to adequately address disparities in care for marginalized communities. Therefore, it is essential to integrate the experiences and needs of these patients into DCC frameworks to ensure more equitable and just treatment [57]. Although the core principles of DCC, including family involvement, psychological support, environmental management, postural support, kangaroo care, breastfeeding, and sleep protection, are well-established, their implementation varies significantly across NICUs in different regions of the world [58,59]. This variability poses challenges to the consistent adoption of DCC principles, potentially affecting the quality of care provided to PNs in each region.

This review underscores the benefits that DCC offers, both in the care of PNs and to their families. However, further research is needed to refine and tailor this care model to the unique circumstances of each country. In this context, it is crucial to thoughtfully adapt DCC programs by taking into account the specific conditions of each institution and the needs of its population [58,59]. To promote the equitable implementation of DCC in NICUs, it is essential to develop culturally responsive interventions, provide language interpretation services, and offer targeted support for families from marginalized or low-income backgrounds [60].

Additionally, healthcare systems should adopt policies that reduce structural barriers, such as transportation support, flexible visiting hours, and on-site parental accommodations, to ensure that all families can actively participate in their infant’s care. These strategies are critical to fostering equitable family engagement and improving neurodevelopmental outcomes in PNs [60].

This review has limitations. First, methodological heterogeneity, variations in participant characteristics, and issues of representativeness make it challenging to draw definitive conclusions and generalize the findings. Despite extensive efforts to include all eligible studies, some relevant research may have been inadvertently overlooked. Moreover, the exclusion of studies focusing on specific pathologies associated with neurodevelopment or exploring healthcare professionals’ perspectives on DCC may limit insights into real-world implementation challenges and facilitators.

Nevertheless, the findings of this review are significant as they offer synthesized, evidence-based insights into the benefits of DCC for the development of PNs. These results provide a solid foundation for future research. In this context, we consider this line of investigation promising, as it contributes to enhancing the quality of care provided to PNs and their families in the NICU.

Additionally, the findings highlight the importance of integrating DCC into routine clinical practice. The neuroprotective strategies identified hold substantial potential to support the neurological development of PNs, fostering comprehensive, family-centered care that prioritizes the well-being of the mother–infant dyad and their families.

## 5. Conclusions

This scoping review identified and synthesized the main interventions associated with DCC for preterm infants in NICUs. The evidence highlights that DCC integrates strategies such as kangaroo care, family-centered care, and NIDCAP, which collectively contribute to improved neurodevelopmental, clinical, and psychosocial outcomes. The primary interventions identified include skin-to-skin contact, specialized preterm feeding practices, and active parental engagement in the caregiving process during hospitalization. These results support the integration of DCC approaches into clinical practice and neonatal care protocols to minimize stimuli that may hinder neurological development in preterm infants.

Despite proven benefits, DCC implementation remains inconsistent across healthcare settings, often limited by structural, socioeconomic, and cultural barriers. Promoting interdisciplinary collaboration and meaningful family engagement is essential to delivering family-centered care that improves both short- and long-term outcomes for preterm infants. Future research should focus on evaluating DCC implementation strategies in diverse contexts, with particular attention to equity, cultural responsiveness, and support for families from marginalized backgrounds to ensure universally accessible, high-quality developmental care.

## Figures and Tables

**Table 1:** General characteristics of studies addressing interventions related to developmentally supportive care for premature newborns.

Author/Year Reference	Language	Country	Study Design	Preterm Newborn’s Gestational Age/Birth Weight	* Level of Evidence	Main Findings
Brignoni-Pérez, et al. 2022 [25]	English	United States	Retrospective observational study	Mean GA: 28.5 ± 2.5 weeks; No BW reported	V	Skin-to-skin contact was lower in infants from families with a lower socioeconomic status or whose families spoke a language other than English.
Franck et al. 2022 [26]	English	United States	Quasi-experimental study	GA: 22–33 weeks; mean BW: ~1190 g	III	Infants whose parents actively participated in mobile integrated family care (mFICare) showed better weight gain and fewer hospital-acquired infections.
Benzies et al. 2020 [27]	English	Canada	Randomized controlled trial	GA: 32.0–34.6 weeks; mean BW: ~2163 g	II	The Alberta integrated family-centered care model in neonatal intensive care units reduced the length of hospital stay in preterm infants, without concomitant increases in readmissions or emergency room visits.
Saldanha and Tauro 2024 [27]	English	India	Quasi-experimental study	Mean GA 27.9 ± 4.6 weeks; BW: 1403 ± 381 g	III	Training in interventions from the newborn individualized developmental care and assessment program improved mothers’ competencies towards their premature neonates.
Gao et al. 2024 [28]	English	China	Retrospective observational study	GA: 22.29–27.86 weeks; BW: 450–1370 g	V	Breastfeeding promotes motor and neurological development in extremely preterm infants, reducing the risk of ventricular hemorrhage.
Pavlyshyn et al. 2023 [29]	English	Ukraine	observational design	GA: 24–32 weeks; BW: 1015–1800 g	V	Developmental care improves early outcomes in extremely and very preterm neonates. Key components include the kangaroo mother care method, stress and pain management, and parental involvement.
Lv et al. 2019 [30]	English	China	Quasi-experimental study	Mean GA: 28.9 ± 1.6 vs. 29.4 ± 2.3 weeks; BW: ~1164–1204 g	III	Very low birth weight preterm infants may experience better clinical health outcomes when parents are present.
Pineda et al. 2018 [31]	English	United States	Prospective cohort study	Mean GA: 28.3 ± 2.7 weeks; BW not reported	IV	Increased parental contact in the neonatal intensive care unit was associated with improved neurobehavioral outcomes prior to discharge. More extensive skin-to-skin care was linked to better gross and fine motor skills at 4–5 years of age.
Kommers et al. 2018 [32]	English	The Netherlands	Non-randomized controlled study	Mean GA: 29.0 weeks; BW: 1267 g	III	Unlike kangaroo care, a mattress designed to mimic the movement of breathing and the sounds of a heartbeat does not affect the heart rate variability of preterm newborns.
Alsadaan et al. 2023 [33]	English	Saudi Arabia	Quasi-experimental study	Mean GA: 28.5 vs. 29.2 weeks; BW: 1250 vs. 1300 g	III	Integrating family-centered care and developmental care in neonatal care improves neurodevelopmental outcomes and reduces hospitalization in high-risk neonates compared to standard care.
Srinath et al. 2016 [34]	English	Canada	Randomized controlled clinical trial	GA: 25–33 weeks; BW: 690–1410 g	II	No significant differences were identified in the physiological and stress responses following the implementation of the kangaroo mother method or the kangaroo father method in preterm neonates.
Yakobson et al. 2021 [35]	English	Israel	Randomized controlled clinical trial	Mean GA: 30.6 ± 2.7 vs. 31.1 ± 2.9 weeks; BW: ~1475–1492 g	II	Music therapy added to skin-to-skin care resulted in greater stability of the autonomic nervous system in preterm neonates.
Gere, Berhane, Worku 2021 [36]	English	Ethiopia	Randomized controlled clinical trial	Mean GE: 33.7 ± 1.3 weeks; BW: 1466 ± 202 g	II	No evidence was found that kangaroo care based on back-to-chest skin-to-skin contact was inferior to chest-to-chest skin-to-skin contact in the regulation of temperature in low birth weight and preterm infants in this trial.
Jamehdar et al. 2022 [37]	English	Iran	Randomized controlled clinical trial	GA: 32–35 weeks; BW not reported	II	When the mother is unable to provide kangaroo care, this type of care can be provided by a surrogate mother, who has been shown to be as effective as the biological mother in improving arterial oxygen saturation and feeding behavior in premature neonates.
Buil et al. 2020 [38]	English	France	Prospective case-control study	Mean GA: 29.7 ± 2.7 vs. 30.0 ± 1.24 weeks; BW: ~1080–1184 g	IV	Supported diagonal flexion positioning creates more opportunities for communication between the mother and the infant during skin-to-skin contact.
Chaudhari et al. 2023 [39]	English	India	Descriptive study	Mean GA: 33.05 ± 1.68 weeks; BW: 1698 ± 495 g	V	Maternal kangaroo care improves cerebral blood flow and stabilizes cardiorespiratory parameters in hemodynamically stable preterm neonates, promoting their physiological stability.
El-Farrash et al. 2020 [40]	English	Egypt	Randomized controlled clinical trial	Mean GA: 32.3–32.5 weeks; BW: 1663–1700 g	II	Preterm neonates who receive kangaroo care for extended periods achieve full enteral feeding more rapidly, experience greater success in breastfeeding, and demonstrate improved neurobehavioral performance, thermal regulation, and tissue oxygenation.
Mirnia et al. 2017 [41]	English	Iran	Randomized controlled clinical trial	Mean GA: 31.4–32.0 weeks; BW: 1788–1906 g	II	The reduction in cortisol levels in the skin-to-skin care group was greater than in the control group, although without significant differences. Therefore, it is possible for parents to care for their infants in an effective, beneficial, and safe manner.
Liang et al. 2022 [42]	English	China	Retrospective observational study	Mean GA: 30.03 ± 1.38 weeks; BW: 1539 ± 334 g	V	Compared to the traditional nursing model, family-centered care in the NICU significantly enhances physical growth and language development in preterm infants.

* System proposed by Melnyk and Fineout-Overholt [24]: Level II—Randomized controlled trials; Level III—Controlled studies without randomization, systematic reviews, or mixed-methods intervention studies; Level IV—Case-control studies or cohort studies; Level V—Systematic reviews of descriptive and qualitative studies. Abbreviations: GA = gestational age; BW = birth weight; SSC = skin-to-skin contact; KMC = kangaroo mother care; FCC = family-centered care; DC = developmental care; NICU = neonatal intensive care unit.

**Table 2:** Interventions Described in the Studies Addressing Developmental Care for Preterm Infants.

Author/Year Reference	Strategy	Main Interventions
Brignoni-Pérez et al. 2022 [25]	Kangaroo care method	Skin-to-skin contact
Franck et al. 2022 [26]	Family-centered care	Breastfeeding Skin-to-skin contact Positive sensory stimulation Pain management through massage Parental education and support
Benzies et al. 2020 [43]	Family-centered care	Alberta FICare™ integrated family care model Relational communication and role negotiation between parents and healthcare professionals Parent education, including individual teaching and group sessions Postpartum depression screening, referrals for psychological support, and assistance from family mentors providing peer support
Saldanha, Tauro 2024 [27]	Individualized developmental care and assessment program for newborns	Communication with parents Newborn safety Newborn feeding Newborn positioning Kangaroo care Infection prevention Newborn skin care
Gao et al. 2024 [28]	Feeding	Breastfeeding
Pavlyshyn et al. 2023 [29]	Family-centered care/kangaroo care method	Control of lighting in the incubator and neonatal unit Gentle and slow handling during clinical management to avoid overstimulation of the newborn Proper positioning of the newborn to ensure comfortable and supportive posture for physical development Grouping interventions to minimize the amount of handling and stress exposure for the baby Involvement of parents in newborn care Skin-to-skin contact Feeding
Lv et al. 2019 [30]	Family-centered care	Theoretical education for parents on basic care, child development, hand hygiene, feeding methods, skin-to-skin contact, and infection control Parental involvement in baby bathing, diaper changing, temperature measurement, and other basic care activities Promotion of breastfeeding among parents Skin-to-skin contact Maternal skill assessment Training of nurses in family-centered care
Pineda et al. 2018 [31]	Kangaroo care method	Skin-to-skin contact
Kommers et al. 2018 [32]	Kangaroo care method	Skin-to-skin contact
Alsadaan et al. 2023 [33]	Family-centered care	Active parental/family involvement in care planning and bedside care Positioning Clustered procedure care Modification of the environment in the neonatal intensive care unit Family education and psychosocial support
Srinath et al. 2016 [34]	Kangaroo care method	Skin-to-skin contact
Yakobson et al. 2021 [35]	Kangaroo care method	Music therapy Skin-to-skin contact
Gere; Berhane; Worku 2021 [36]	Kangaroo care method	Skin-to-skin contact
Jamehdar et al. 2022 [37]	Kangaroo care method	Skin-to-skin contact
Buil et al. 2020 [38]	Kangaroo care method	Positioning Skin-to-skin contact
Chaudhari et al. 2023 [39]	Kangaroo care method	Positioning Skin-to-skin contact Breastfeeding
El-Farrash et al. 2020 [40]	Kangaroo care method	Skin-to-skin contact Breastfeeding
Mirnia et al. 2017 [41]	Kangaroo care method	Skin-to-skin contact
Liang et al. 2022 [42]	Family-centered care	Parental involvement in newborn care Adjusting newborn’s body position Diaper changes and estimation of urine volume Umbilical cord care Oral care Skin-to-skin kangaroo contact Psychological support for parents Communication between parents and healthcare staff during daily rounds regarding the newborn’s current situation and treatment plan

## Data Availability

The original contributions presented in the study are included in the article; further inquiries can be directed to the corresponding author.

## Abbreviations

The following abbreviations are used in this manuscript:

WHO	World Health Organization
PN	preterm newborn
NICU	neonatal intensive care unit
FCCM	family-centered care model
IDCM	integrative developmental care model
DCC	developmental-centered care
KCM	kangaroo care method
NIDCAP	neonatal individualized developmental care and assessment program
SDGs	Sustainable Development Goals
RCT	randomized controlled trials

## References

[1] World Health Organization (2022). Preterm Births.

[2] Soleimani F., Azari N., Ghiasvand H., Shahrokhi A., Rahmani N., Fatollahierad S. (2020). Do NICU developmental care improve cognitive and motor outcomes for preterm infants? A systematic review and meta-analysis. BMC Pediatr..

[3] Ohuma E.O., Moller A.-B., Bradley E., Chakwera S., Hussain-Alkhateeb L., Lewin A., Okwaraji Y.B., Mahanani W.R., Johansson E.W., Lavin T. (2023). National, regional, and global estimates of preterm birth in 2020, with trends from 2010: A systematic analysis. Lancet.

[4] Gómez-Cantarino S., García-Valdivieso I., Moncunill-Martínez E., Yáñez-Araque B., Gurrutxaga M.I.U. (2020). Developing a family-centered care model in the neonatal intensive care unit (Nicu): A new vision to manage healthcare. Int. J. Environ. Res. Public Health.

[5] Kara Ö.K., Kara K., Kara K., Arslan M. (2020). Neuromotor and sensory development in preterm infants: Prospective study. Turk. Arch. Pediatr..

[6] Raghupathy M.K., Rao B.K., Nayak S.R., Spittle A.J., Parsekar S.S. (2021). Effect of family-centered care interventions on motor and neurobehavior development of very preterm infants: A protocol for systematic review. Syst. Rev..

[7] Aita M., Faugère G.D.C., Lavallée A., Feeley N., Stremler R., Rioux É., Proulx M.-H. (2021). Effectiveness of interventions on early neurodevelopment of preterm infants: A systematic review and meta-analysis. BMC Pediatr..

[8] Browne J.V., Jaeger C.B., Kenner C. (2020). Executive summary: Standards, competencies, and recommended best practices for infant- and family-centered developmental care in the intensive care unit. J. Perinatol..

[9] Altimier L., Kenner C., Damus K. (2015). The Wee Care Neuroprotective NICU Program (Wee Care): The Effect of a Comprehensive Developmental Care Training Program on Seven Neuroprotective Core Measures for Family-Centered Developmental Care of Premature Neonates. Newborn Infant Nurs. Rev..

[10] Cardin A.D., Rens L., Stewart S., Danner-Bowman K., McCarley R., Kopsas R. (2015). Neuroprotective Core Measures 1–7: A Developmental Care Journey: Transformations in NICU Design and Caregiving Attitudes. Newborn Infant Nurs. Rev..

[11] Almadhoob A., Ohlsson A. (2020). Sound reduction management in the neonatal intensive care unit for preterm or very low birth weight infants. Cochrane Database Syst. Rev..

[12] Charpak N., Montealegre-Pomar A., Bohorquez A. (2020). Systematic review and meta-analysis suggest that the duration of Kangaroo mother care has a direct impact on neonatal growth. Acta Paediatr..

[13] Gupta N., Deierl A., Hills E., Banerjee J. (2021). Systematic review confirmed the benefits of early skin-to-skin contact but highlighted lack of studies on very and extremely preterm infants. Acta Paediatr..

[14] Chandebois L., Nogue E., Bouschbacher C., Durand S., Masson F., Mesnage R., Nagot N., Cambonie G. (2021). Dissemination of newborn behavior observation skills after Newborn Individualized Developmental Care and Assessment Program (NIDCAP) implementation. Nurs. Open.

[15] Phillips R.M. (2015). Seven Core Measures of Neuroprotective Family-Centered Developmental Care: Creating an Infrastructure for Implementation. Newborn Infant Nurs. Rev..

[16] Soni R., Wel-Wel C.T., Robertson N.J. (2021). Neuroscience meets nurture: Challenges of prematurity and the critical role of family-centred and developmental care as a key part of the neuroprotection care bundle. Arch. Dis. Child.-Fetal Neonatal Ed..

[17] Lawn J.E., Bhutta Z.A., Ezeaka C., Saugstad O. (2023). Ending Preventable Neonatal Deaths: Multicountry Evidence to Inform Accelerated Progress to the Sustainable Development Goal by 2030. Neonatology.

[18] Lucas J.E., Richter L.M., Daelmans B. (2017). Care for Child Development: An intervention in support of responsive caregiving and early child development. Child Care Health Dev..

[19] Dua T., Tomlinson M., Tablante E., Britto P., Yousfzai A., Daelmans B., Darmstadt G.L. (2016). Global research priorities to accelerate early child development in the sustainable development era. Lancet Glob. Health.

[20] Lockwood C., dos Santos K.B., Pap R. (2019). Practical Guidance for Knowledge Synthesis: Scoping Review Methods. Asian Nurs. Res..

[21] Tricco A.C., Lillie E., Zarin W., O’Brien K.K., Colquhoun H., Levac D., Moher D., Peters M.D.J., Horsley T., Weeks L. (2018). PRISMA extension for scoping reviews (PRISMA-ScR): Checklist and explanation. Ann. Intern. Med..

[22] Ouzzani M., Hammady H., Fedorowicz Z., Elmagarmid A. (2016). Rayyan—A web and mobile app for systematic reviews. Syst. Rev..

[23] Peters M.D.J., Marnie C., Tricco A.C., Pollock D., Munn Z., Alexander L., McInerney P., Godfrey C.M., Khalil H. (2020). Updated methodological guidance for the conduct of scoping reviews. JBI Évid. Synth..

[24] Melnyk B.M., Fineout-Overholt E. (2011). Melnyk Pyramid: Levels of Evidence. Evidence-Based Practice in Nursing and Healthcare: A Guide to Best Practice. https://books.google.es/books/about/Evidence_based_Practice_in_Nursing_Healt.html?id=hHn7ESF1DJoC&redir_esc=y.

[25] Brignoni-Pérez E., Scala M., Feldman H.M., Marchman V.A., Travis K.E. (2021). Disparities in Kangaroo Care for Premature Infants in the Neonatal Intensive Care Unit. J. Dev. Behav. Pediatr..

[26] Franck L.S., Gay C.L., Hoffmann T.J., Kriz R.M., Bisgaard R., Cormier D.M., Joe P., Lothe B., Sun Y. (2022). Neonatal outcomes from a quasi-experimental clinical trial of Family Integrated Care versus Family-Centered Care for preterm infants in U.S. NICUs. BMC Pediatr..

[27] Saldanha S.J., Tauro V.G. (2023). Competency based performance of mothers on preterm neonatal care through Neonatal Integrative Developmental Care (NIDC) interventions: An interventional pilot project. J. Neonatal Nurs..

[28] Gao Y., Lu X., Pan M., Liu C., Min Y., Chen X. (2024). Effect of breast milk intake volume on early behavioral neurodevelopment of extremely preterm infants. Int. Breastfeed. J..

[29] Pavlyshyn H., Sarapuk I., Tscherning C., Slyva V. (2022). Developmental care advantages in preterm infants management. J. Neonatal Nurs..

[30] Lv B., Gao X.-R., Sun J., Li T.-T., Liu Z.-Y., Zhu L.-H., Latour J.M. (2019). Family-centered care improves clinical outcomes of very-low-birth-weight infants: A quasi-experimental study. Front. Pediatr..

[31] Pineda R., Bender J., Hall B., Shabosky L., Annecca A., Smith J. (2018). Parent participation in the neonatal intensive care unit: Predictors and relationships to neurobehavior and developmental outcomes. Early Hum. Dev..

[32] Kommers D., Joshi R., van Pul C., Feijs L., Oei G., Oetomo S.B., Andriessen P. (2018). Unlike Kangaroo care, mechanically simulated Kangaroo care does not change heart rate variability in preterm neonates. Early Hum. Dev..

[33] Alsadaan N., Ramadan O.M.E., Alqahtani M., Shaban M., Elsharkawy N.B., Abdelaziz E.M., Ali S.I. (2023). Impacts of Integrating Family-Centered Care and Developmental Care Principles on Neonatal Neurodevelopmental Outcomes among High-Risk Neonates. Children.

[34] Srinath B.K., Shah J., Kumar P., Shah P.S. (2015). Kangaroo care by fathers and mothers: Comparison of physiological and stress responses in preterm infants. J. Perinatol..

[35] Yakobson D., Gold C., Beck B.D., Elefant C., Bauer-Rusek S., Arnon S. (2021). Effects of live music therapy on autonomic stability in preterm infants: A cluster-randomized controlled trial. Children.

[36] Gere S., Berhane Y., Worku A., Nimbalkar S.M. (2021). Chest-to-Back Skin-to-Skin Contact to Regulate Body Temperature for Low Birth Weight and/or Premature Babies: A Crossover Randomized Controlled Clinical Trial. Int. J. Pediatr..

[37] Jamehdar M., Nourizadeh R., Divband A., Valizadeh L., Hosseini M., Hakimi S. (2022). KMC by surrogate can have an effect equal to KMC by mother in improving the nutritional behavior and arterial oxygen saturation of the preterm infant: Results of a controlled randomized clinical trial. BMC Pediatr..

[38] Buil A., Sankey C., Caeymaex L., Apter G., Gratier M., Devouche E. (2020). Fostering mother-very preterm infant communication during skin-to-skin contact through a modified positioning. Early Hum. Dev..

[39] Chaudhari A.J., Nimbalkar S.M., Patel D.V., Phatak A.G. (2022). Effect of Kangaroo Mother Care on Cerebral Hemodynamics in Preterm Neonates Assessed by Transcranial Doppler Sonography in Middle Cerebral Artery. Indian Pediatr..

[40] El-Farrash R.A., Shinkar D.M., Ragab D.A., Salem R.M., Saad W.E., Farag A.S., Salama D.H., Sakr M.F. (2019). Longer duration of kangaroo care improves neurobehavioral performance and feeding in preterm infants: A randomized controlled trial. Pediatr. Res..

[41] Mirnia K., Bostanabad M.A., Asadollahi M., Razzaghi M.H. (2017). Paternal skin-to-skin care and its effect on cortisol levels of the infants. Iran. J. Pediatr..

[42] Liang X., Miao A., Zhang W., Li M., Xing Y. (2022). Effect of family integrated care on physical growth and language development of premature infants: A retrospective study. Transl. Pediatr..

[43] Benzies K.M., Aziz K., Shah V., Faris P., Isaranuwatchai W., Scotland J., Larocque J., Mrklas K.J., Naugler C., Stelfox H.T. (2020). Effectiveness of Alberta Family Integrated Care on infant length of stay in level II neonatal intensive care units: A cluster randomized controlled trial. BMC Pediatr..

[44] Lisseth B.C., Alejandra M.P., Coo S. (2021). Developmental care of premature newborns: Fundamentals and main characteristics. Andes Pediatr..

[45] Vohr B., McGowan E., McKinley L., Tucker R., Keszler L., Alksninis B. (2017). Differential Effects of the Single-Family Room Neonatal Intensive Care Unit on 18- to 24-Month Bayley Scores of Preterm Infants. J. Pediatr..

[46] World Health Organization (2021). Immediate “Kangaroo Mother Care” and Survival of Infants with Low Birth Weight. N. Engl. J. Med..

[47] World Health Organization (2023). Kangaroo Mother Care Implementation Strategy for Scale-Up Adaptable to Different Country Contexts.

[48] Sarin E., Maria A. (2019). Acceptability of a family-centered newborn care model among providers and receivers of care in a Public Health Setting: A qualitative study from India. BMC Health Serv. Res..

[49] Klein V., Zores-Koenig C., Dillenseger L., Langlet C., Escande B., Astruc D., Le Ray I., Kuhn P., Strasbourg NIDCAP Study group (2021). Changes of Infant- and Family-Centered Care Practices Administered to Extremely Preterm Infants During Implementation of the NIDCAP Program. Front. Pediatr..

[50] Ohlsson A., Jacobs S.E. (2013). NIDCAP: A Systematic Review and Meta-analyses of Randomized Controlled Trials. Pediatrics.

[51] Wallace L.S., Okito O., Mellin K., Soghier L. (2024). Associations between Parental Engagement in the Neonatal Intensive Care Unit and Neighborhood-Level Socioeconomic Status. Am. J. Perinatol..

[52] Sigurdson K., Profit J., Dhurjati R., Morton C., Scala M., Vernon L., Randolph A., Phan J.T., Franck L.S. (2020). Former NICU Families Describe Gaps in Family-Centered Care. Qual. Health Res..

[53] Cai Q., Chen D.-Q., Wang H., Zhang Y., Yang R., Xu W.-L., Xu X.-F. (2022). What influences the implementation of kangaroo mother care? An umbrella review. BMC Pregnancy Childbirth.

[54] Vittner D., Butler S., Lawhon G., Buehler D. (2024). The newborn individualised developmental care and assessment program: A model of care for infants and families in hospital settings. Acta Paediatr..

[55] Chan G.J., Labar A.S., Wall S., Atun R. (2015). Kangaroo mother care: A systematic review of barriers and enablers. Bull. World Health Organ..

[56] Yue J., Liu J., Williams S., Zhang B., Zhao Y., Zhang Q., Zhang L., Liu X., Wall S., Wetzel G. (2020). Barriers and facilitators of kangaroo mother care adoption in five Chinese hospitals: A qualitative study. BMC Public Health.

[57] Sigurdson K., Morton C., Mitchell B., Profit J. (2018). Disparities in NICU quality of care: A qualitative study of family and clinical accounts. J. Perinatol. Off. J. Calif. Perinat. Assoc..

[58] Lopez-Maestro M., De la Cruz J., Perapoch-Lopez J., Gimeno-Navarro A., Vazquez-Roman S., Alonso-Diaz C., Muñoz-Amat B., Morales-Betancourt C., Soriano-Ramos M., Pallas-Alonso C. (2019). Eight principles for newborn care in neonatal units: Findings from a national survey. Acta Paediatr. Int. J. Paediatr..

[59] Roué J.-M., Kuhn P., Maestro M.L., Maastrup R.A., Mitanchez D., Westrup B., Sizun J. (2017). Eight principles for patient-centred and family-centred care for newborns in the neonatal intensive care unit. Arch. Dis. Child.-Fetal Neonatal Ed..

[60] Lechner B.E., Kukora S.K., Hawes K. (2024). Equity, inclusion and cultural humility: Contemporizing the neonatal intensive care unit family-centered care model. J. Perinatol..

